# Supplementary material for: Data quality assessment and associated factors in the health management information system among health centers of Southern Ethiopia
Source: PLoS One. 2021 Oct 27;16(10):e0255949. doi: 10.1371/journal.pone.0255949 (PMC8550403; doi:10.1371/journal.pone.0255949)
Supplement: S1 File — (DOCX) [file pone.0255949.s001.docx]

# Questionnaire English version

**Part** one Routine Data quality Assessment: Health Facility tool

| **NOTE TO THE INTERVIEWER:** explain the tool to the person who will serve as a facilitator for record review and you will have to visit different departments within the health center | | |
| --- | --- | --- |
| 01 | ________/_________/_______________  DD / MM / YYYY | |
| Health center Identification | | |
| Woreda | |  |
| Name of the Health center | |  |
| Telephone Number (Office) | |  |
| Position of person interviewed | |  |

| General information | | |
| --- | --- | --- |
| 1. Is there designated staff (HMIS focal person) responsible  For reviewing aggregated numbers prior to submission to  the next level (e.g., to districts ) | 1. Yes | 2.No |
| **2.** The responsibility for recording the delivery of services on source documents is clearly assigned (have written job descriptions)to the relevant staff | **1.** Yes | 2.No |
| 4. Does the health center have electronic data base (computer software)? (If no, skip to 7) | 1. Yes | 2.No |
| 5. Is the electronic database (computer software) currently functional? | 1. Yes | 2.No |
| 6. Is there any system in place to prevent unauthorized changes to data? | 1. Yes | 2.No |
| 7.The service delivery point establish performance monitoring team | 1.Yes | 2.No |

| Data Recording | | | | |
| --- | --- | --- | --- | --- |
| 1 | Does this Health center keep copies of the RHIS monthly reports which are sent to the district office? | 1. Yes | 2.No | If no go to Q5 |
| 2 | Count the number of RHIS monthly reports that have been kept at the facility for the last twelve months |  |  |  |
| 3 | Does this health center keep an outpatient register? | 1.Yes | 2.No | If no go to Q5 |

| Record review tool to assess Data Accuracy | | | | | |  |
| --- | --- | --- | --- | --- | --- | --- |
| 9. Find the following information from registers for the selected two months. Compare the figures with the paper based data base submitted to next level | | | | | |  |
| If one or more of the following services are not provided in the institution, please include a replacement data element | | |  | | |  |
| Item | Data elements | # from registers | # from report submitted | # from registers | # from report submitted |  |
| 9.1 | Antenatal care fourth visits (ANC4) |  |  |  |  |  |
| 9.2 | Total no of births attended by skilled personnel (institutional deliveries) |  |  |  |  |  |
| 9.3 | Under one yrs who take Pentavalent third doses (check EPI records) |  |  |  |  |  |
| 9.4 | PMTCT |  |  |  |  |  |
| 9.5 | Tuberculosis cure rate(PTB) |  |  |  |  |  |
| 9.6 | Total Contraceptive accepters(repeat and new) |  |  |  |  |  |
| 9.7 | Confirmed malaria cases |  |  |  |  |  |

| Record review tool to assess content Completeness from 2 months report | | | |  |
| --- | --- | --- | --- | --- |
| 10 | How many data items does the heath center need to report on in the RHIS monthly report? This number does not include data items for services not provided by this health center |  |  |  |
| 11 | Count the number of data items that are supposed to be filled in by this facility but left blank without indicating “0” in the selected month’s report. |  |  |  |

**Part two; - Organizational and behavioural Assessment Tool**

Self -administered questionnaire for health workers

| **For the interviewer;**-give information about the study and ask for consent to participate in the study | | |
| --- | --- | --- |
| 01 | ________/_________/_______________  DD / MM / YYYY | |
| Facility Identification | | |
| Woreda | |  |
| Name of Health center | |  |
| Department | |  |
| Telephone Number (Office) | |  |

101. Sex 1. Male 2. Female

102. Age of the respondent in years …………………………

103. Position of person interviewed

1. Head of institution

2. Department Head

3. HMIS Focal Person

4. Other (specify) ----------------

104. Highest Level of Education

1. Level 3/Certificate

2. Level 4/Diploma

3. Bachelor Degree

4. Master Degree

5. Other (specify) _______

105. Field of study for the highest level of education

1. Nurse

2. Midwife

3. Health Officer

4. Laboratory Technology

5. Health information& technology ( HIT)

6. Pharmacist

7. Other (specify)

106. Years of experience ____________

107. Have you ever received in-service training on HMIS related activities in last six months?

1. Yes 2. No

108. Did you receive pre-service training on HMIS? 1. Yes 2. No

109. Have you been participated in aggregation or compilation of data from tally Sheet/registration? 1. Yes 2. No

110. Do you think that registrations and report formats are user friendly /easily understandable?

1. Yes 2. No.

111. Do you register all your activity on daily basis? 1. Yes 2. No

112. Do you fill the register /tally sheet completely? 1. Yes 2. No

113. Is the report submitted complete, timely, and accurate? 1. Yes 2. No

114. Did you conduct data accuracy test? 1. Yes 2. No

115. If yes, How frequently? 1. Monthly 2. Quarterly 3. Semi-annually 4.annually

116. In the past 3 months did you get supervision from higher officials?

1. Yes 2. No

117. If yes for Q14, how many times the unit/department supervised.

1. One time 2. Two times 3. Three times

118. Did you get regular Feedback from top level organization through regular report based on evidence ? 1. Yes 2. No

119. If yes, how often? 1. Monthly 2. Quarterly 3. Semi-annually 4. Annually

120. Do you have standard set of indicators with their definition? 1. Yes 2. No

121. Do you have procedural manual in your department? 1. Yes 2. No

122. Are there any incentives for HMIS process? 1. Yes 2. No

123. If yes, what kind of incentives 1. Training 2. Money 3. Recognition 4. Other (specify )

124. Is there skilled human resource able to fill formats? 1. Yes 2. No

**INSTRUCTIONS**

We would like to know your opinion about how strongly you agree with certain activities carried out by _______________. There are no right or wrong answers, but only expression of your opinion on a scale. The scale is about assessing the intensity of your belief and ranges from strongly disagree (1) to strongly agree (5).

You have to determine first whether you agree or disagree with the statement. Second decide about the intensity of agreement or disagreement.

If you **disagree** with statement then use left side of the scale and determine how much disagreement that is strongly disagree (1) or disagree (2) and circle the appropriate answer. If you are not sure of your belief or think that you neither disagree nor agree, then circle 3.

If you **agree** with the statement, then use right side of the scale and determine how much agreement that is agree (4) or strongly agree (5) and circle the appropriate answer.

Please note that you might agree or disagree with all the statements and similarly you might not have the same intensity of agreement or disagreement and thus variations are expected in expressing your agreement or disagreement. We encourage you to express those variations.

This information will remain confidential and would not be shared with anyone, except presented as an aggregated data report. Please be frank and choose your answer honestly.

To what extent, do you agree with the following on a scale of 1-5

| knowledge of HMIS | Strongly disagree  (1) | Disagree (2) | Neutral (3) | Agree (4) | Strongly Agree (5) |
| --- | --- | --- | --- | --- | --- |
| 1.HMIS Collects data from service and administrative records |  |  |  |  |  |
| 2.HMIS Provides signals that can be reviewed frequently to monitor program implementation |  |  |  |  |  |
| 3.HMIS is Used for decision making |  |  |  |  |  |
| 4.HMIS is important for policy Making and management decisions |  |  |  |  |  |
| 5.HMIS is important for Monitoring and Evaluation of performance |  |  |  |  |  |
| 6.HMIS data can be presented by using Charts, graphs and tables |  |  |  |  |  |
| 7.HMIS is an integral part of Health Information System |  |  |  |  |  |

| Questions to assess supervision quality | Strongly disagree  (1) | Disagree (2) | Neutral (3) | Agree (4) | Strongly Agree (5) |
| --- | --- | --- | --- | --- | --- |
| 1. Seek feedback from concerned persons |  |  |  |  |  |
| 2. Emphasize data quality in monthly reports |  |  |  |  |  |
| 3. Discuss conflicts openly to resolve them |  |  |  |  |  |
| 4. Seek feedback from concerned community |  |  |  |  |  |
| 5.Use HMIS data for setting targets and  Monitoring |  |  |  |  |  |
| 6. Check data quality regularly |  |  |  |  |  |
| 7. Provide regular feedback to their staff through regular report based on evidence |  |  |  |  |  |
| 8. Report on data accuracy regularly  (Talk to higher level staff about accuracy of data) |  |  |  |  |  |
| 9. Encourage their supervisees to over report (false report) their performance |  |  |  |  |  |

| In your health center, staffs | | Strongly disagree  (1) | | Disagree (2) | Neutral (3) | Agree (4) | Strongly Agree (5) |
| --- | --- | --- | --- | --- | --- | --- | --- |
| 1. Document their activities and keep records | |  | |  |  |  |  |
| 2. Feel committed in improving health status of the target population | |  | |  |  |  |  |
| 3. Set appropriate and double target of their performance | |  | |  |  |  |  |
| 4. Feel guilty for not accomplishing the set target/performance | |  | |  |  |  |  |
| 5. Are rewarded for good work | |  | |  |  |  |  |
| 6. staffs are empowered to make decisions |  | |  | |  |  |  |
| 7. Able to say no to supervisors and colleagues for demands/decisions not supported by evidence |  | |  | |  |  |  |
| 8. Are made accountable for poor performance |  | |  | |  |  |  |
| 9. Use HMIS data for community education and mobilization |  | |  | |  |  |  |
| 10. Admit mistakes for taking corrective actions |  | |  | |  |  |  |

| Management support | Strongly disagree | | Disagree (2) | | Neutral (3) | Agree (4) | Strongly Agree |
| --- | --- | --- | --- | --- | --- | --- | --- |
| 1.health department Use HMIS data for day to day management of the health center |  | |  | |  |  |  |
| 2. Health department encourages to display data for monitoring their set target |  | |  | |  |  |  |
| 3. Health department encourages to gather data to find the root cause(s) of the problem |  | |  | |  |  |  |
| 4. Health department encourages to develop appropriate criteria for selecting interventions for a given problem |  | |  | |  |  |  |
| 5. Health department encourages to develop appropriate outcomes for a particular intervention |  | |  | |  |  |  |
| 6. Health department encourages to evaluate whether the targets or outcomes have been achieved |  | |  | |  |  |  |
| Motivation | | Strongly disagree  (1) | | Disagree (2) | Neutral (3) | Agree (4) | Strongly Agree (5) |
| 1. Collecting information which is not used for decision making discourages me | |  | |  |  |  |  |
| 2. Collecting information makes me feel bored | |  | |  |  |  |  |
| 3. Collecting information is meaningful for me | |  | |  |  |  |  |
| 4. Collecting information gives me the feeling that data is needed for monitoring facility performance | |  | |  |  |  |  |
| 5. Collecting information give me the Feeling that it is forced on me | |  | |  |  |  |  |
| 6. Collecting information is appreciated by Co-workers and supervisors | |  | |  |  |  |  |

PART THREE;-SELF-EFFICACY

This part of the questionnaire is about your perceived confidence in performing tasks related to health information systems. High Confidence indicates that person could perform the task, while low confidence means room for improvement. We are interested in knowing how confident you feel in performing HMIS related tasks. Please be frank and rate your confidence honestly.

Please rate your confidence in percentages that you can accomplish the HMIS activities.

Rate your confidence for each situation with a percentage from the following scale

0 10 20 30 40 50 60 70 80 90 100

| Self-Efficacy | No | | | | | | Yes | | | | |
| --- | --- | --- | --- | --- | --- | --- | --- | --- | --- | --- | --- |
|  | 0 | 10 | 20 | 30 | 40 | 50 | 60 | 70 | 80 | 90 | 100 |
| 1. I can check data accuracy |  |  |  |  |  |  |  |  |  |  |  |
| 2. I can calculate percentages/rates correctly |  |  |  |  |  |  |  |  |  |  |  |
| 3. I can plot data by months or years |  |  |  |  |  |  |  |  |  |  |  |
| 4. I can compute trend from bar charts |  |  |  |  |  |  |  |  |  |  |  |
| 5. I can explain findings & their implications |  |  |  |  |  |  |  |  |  |  |  |
| 6. I can use data for identifying gaps and setting targets |  |  |  |  |  |  |  |  |  |  |  |
| 7. I can use data for making various types of decisions and providing feedback |  |  |  |  |  |  |  |  |  |  |  |

Thank you for your response and time!
