# Supplementary material for: Data quality assessment and associated factors in the health management information system among health centers of Southern Ethiopia
Source: PLoS One. 2021 Oct 27;16(10):e0255949. doi: 10.1371/journal.pone.0255949 (PMC8550403; doi:10.1371/journal.pone.0255949)
Supplement: S2 File — (DOCX) [file pone.0255949.s002.docx]

**Amharic language version questionnaire**

መረጃ መጠየቂያ ቅፅ

ጤና ይስጥልኝ!

ስሜ………………………….ይባላል፡፡የመጣሁት የአዲስ አበባ ዩኒቨርስቲ ህብረተሰብ ጤና ሳይንስ ተማሪ የሆነችውን ማስተዋል ሰለሞንን ወክዬ ነው፡፡የሁለተኛ ድግሪ መመረቂያ ጥናቷን የምትሰራው የመረጃ አያያዝ ስረአት ጥራት እና ተያያዥ ጉዳዮችን በተመለከተ ነው፡፡ ይህንን ጥናት ለማድረግ ከአዲስ አበባ ዩኒቨርሲቲና ጤና ቢሮ ፈቃድ አግኝታለች፡፡

እርስዎ የተመረጡት በመረጃ ያያዝ ስርአት ላይ ስለሚሰሩ ነው፡፡በጥናቱ ላይ መሳተፍ ሙሉ በሙሉ በርሰዎ ፈቃድ ላይ የተመሰረተ ሲሆን በጥናቱ የመሳተፍ ወይም አለመሳተፍ ሙሉ መብት አለዎት አንዲሁም ለመሳተፍ ፈቃደኛ ከሆኑ በኃላ በፈለጉት ጊዜ ማቋረጥ ወይ ማቆም ይችላሉ፡፡በጥናቱ አለመሳተፍ የሚያደርስቦት ጉዳት የለም፡፡ከዚህ ጥናት የተሰበሰበው መረጃ ሙሉ ሚስጥራዊነቱ የተጠበቀ ይሆናል፡፡ ከጥናት ቡድኑ ውጪ ማንም የተሰበሰበውን መረጃ ማግኘት አይችልም፡፡እንዲሁም መረጃው ከጥናቱ አላማ ውጪ ለምንም አንጠቀምበትም፡፡

በጥናቱ በመሳተፍዎ ለሚሰሩበት ጤና ተቋም የመረጃ አያያዝ ስረዓት ጥራትን ለማማሻሻል ይረዳል፡፡የእርስዎ ፈቃደኝነትና የነቃ ተሳትፎ ለዚህ ጥናት ስኬታማነት አስፈላጊ ነው፡፡

ስለጥናቱ ጥያቄ ወይም ተጨማሪ መረጃ ከፈለጉ በዚህ አድራሻ መጠየቅ ይችላሉ፡፡

ስም፡- ማስተዋል ሰለሞን

ስልክ ቁጥር፡-0926078254

ኢሜል፡-massfiker2012@gmail.com

የስምምነት መጠየቂያ/ማረጋገጫ ቅፅ

ከላይ በተሰጡት መረጃ መሰረት በዚህ ጥናት ለመሳተፍ ፈቃደኛ ኖት

1. አዎ( ቃለመጠይቁ ይቀጥል)
2. አይደለሁም ( አመስግነህ ወደሚቀጥለው ተሳታፊ እለፍ )

ቃለመጠይቅ አድራጊው ስም ፊርማ

ቁጥር

ቃለ መጠይቅ የተካሄደበት ቀን የተጀመረበት ሰዓት ያለቀበት ሰዓት

መጠይቁ ታይቷል/ ተፈትሾል

የቃለ መጠይቁ ውጤት፡ 1.ሙሉ በሙሉ የተሟላ

2. ያልተገኙ

3. ፍቃደኛ ያልሆኑ

4. በከፊል የተሟላ

በተቆጣጣሪዎችተረጋግጧል ስም ፊርማ

- **በአስተዳዳሪዎችና በሰራተኞች የሚሞላ**

| 01 | ________/_________/_______________  ቀን / ወር / አ.ም | |
| --- | --- | --- |
| የጤና ጣቢያው መገለጫ | | |
| ወረዳ | |  |
| የጤና ጣቢያው ስም | |  |
| የስራ ክፍል | |  |
| ስልክ ቁጥር | |  |

101. ጾታ 1.ወንድ 2.ሴት

102. የተጠያቂው እድሜ…………………………

103. ቃለ መጠይቁን የሚሞላው ሰው ማእረግ

1. የጤና ጣቢያው ሀላፊ
2. የክፍሉ ሀላፊ
3. የ HMIS ፎካል
4. አገልግሎት ሰጪ/ ባለሙያ

104. የትምህርት ደረጃ

1. ሌቭል 3/ሰርተፍኬት
2. ሌቭል4/ዲፕሎማ
3. የመጀመሪያ ዲግሪ
4. ሁለተኛ ዲግሪ
5. ሌላ(ይጠቀስ)____

105. የተማሩበት የትምህርት መስክ

- 1. ነርስ
  2. አዋላጅ ነርስ
  3. ጤና መኮንን
  4. የላብራቶሪ ባለሙያ
  5. የጤና መረጃ አያያዝ ባለሙያ
  6. የፋርማሲ ባለሙያ
  7. ሌላ(ይጠቀስ)-------------

106. የስራ ልምድ ____________

107. ባለፉት ስድስት ወራት ውስጥ ከ HMIS ጋ በተያያዘ ስልጠና ወስደው ያውቃሉ?

1. አዎ 2. አልወሰድኩም

108. ወደ ስራ ከመግባትዎ አስቀድመው በHMIS ላይ ስልጠና ወስደዋል?

1. አዎ 2. አልወሰድኩም

109. ከወርሃዊ/ከእለት መዝገብ ላይ መረጃን በመሰብሰብ ወይም በማጠናቀር ተሳትፈው ያውቃሉ?

1. አዎ 2. አላውቅም

110. የ HMIS መዝገቦችና የሪፖርት ፎርማቶች ቀላልና መረዳት የሚቻሉ ናቸው ?

1. አዎ 2. አይደሉም

111. ሁሉንም ስራዎችዎን በየለቱ ይመዘግባሉ ? 1. አዎ 2. አይደለም

112. ወርሃዊ/እለት መዝገቦችን ጨርሰው/አማልተው ይመዘግባሉ ?

1. አዎ 2. አይደለም

113. የሚያስገቡት ሪፖርት የተማላ፤ጊዜውን የተበቀና ትክክለኛ ነው ?

114. የመረጃን ትክክለኛነት አረጋግጠው(LQAS) ያውቃሉ?

1. አዎ 2. አይደለም

115. መልስዎ አዎ ከሆነ በምን ያህል ጊዜ ?

1. በየወሩ 2. በየሶስት ወር 3. በየስድስት ወር 4.በየአመቱ

116.ባለፉት ሶስት ወራት ውሰጥ ከበላይ አለቆች ድጋፋዊ ክትትል አግኝተው ያውቃሉ ?

1. አዎ 2. አይደለም

117. መልስዎ አዎ ከሆነ በምን ያህል ጊዜ ?

1. አንድ ጊዜ 2. ሁለት ጊዜ 3. ሶሰት ጊዜ

118. ከበላይ አለቆች በጽሁፍ የተደገፈ ግብረ-መልስ አግኝተው ያውቃሉ ?

1. አዎ 2. አይደለም

119.መልስዎ አዎ ከሆነ በምን ያህል ጊዜ ?

1.በየወሩ 2. በየሶስት ወር 3. በየስድስት ወር 4.በየአመቱ

120. በሚሰሩበት ጤና ጣቢያ የ HMIS ጠቆሚዎችን የያዙ ስብስቦች አሉ ?

1. አዎ 2. አይደለም

121. በሚሰሩበት ጤና ጣቢያ የመረጃ አሰባሰብ ስርዓት ማኑዋሎች አሉ ?

1. አዎ 2. አይደለም

122 በሚሰሩበት ጤና ጣቢያ ከ ጋር በተያያዘ የሚሰጥ ማበረታቻ አለ?

1. አዎ 2. አይደለም

123. መልስዎ አዎ ከሆነ በምን አይነት?

1. ገንዘብ 2. ስልጠና 3. እውቅና 4. ሌላ(ይጠቀስ)---------

124. በሚሰሩበት ጤና ጣቢያ መዝገቦችንና ፎርማቶችን ለመሙላት የሚችሉ የሰለጠኑ ባለሙያዎች አሉ ?

1. አዎ 2. አይደለም

- በጤና ቋሙ ከተሰሩት ስራዎች ጋር ምን ያህል እንደሚስማሙ የርስዎን አመለካከት ማወቅ እንፈልጋለን፡፡

በመለኪያው የርስዎን አመለካከት ከመግለጽ በዘለለ ትክክልና ትክክል ያልሆኑ መልሶች የሉም፡፡መለኪያው የርስዎን የአመለካከት ጥንካሬ የሚመዝን ሲሆን ከፍፁም አልስማማም(1) እሰከ ፍፁም እስማማለሁ(5) ምርጫዎች ይሰጣል፡፡ አመለካከትዎን በበለጠ ይገልፃል የሚሉትን ምርጫ በማክበብ እንዲያመለክቱ እንጠይቃለን፡ምንም እንክዋን ከሁሉም ሃሳቦች ጋር ሊስማሙ ወይም ላይስማሙ ቢችሉም ለሁሉም አንድ አይነት የመስማማትና ያለማስስማማት ጥንካሬ እንደማይኖርዎትና ልዩነቶች ሊኖሩ እንደሚችሉ ይጠበቃል፡፡እነዚህ ልዩነቶች ጎልተው እንዲወጡ እንፈለጋለን፡፡የሚሰጡንን መረጃዎች በሚስጥር የሚያዙና ለሶስተኛ ወገን ተላልፈው እንደማይሰጡ ልንገልፅልዎ እንወዳለን፡፡መልስዎን በሀቀኝነት እንደሚሰጡን እምነታችን ነው፡፡

ከሚከተሉት ነጥቦች ጋር ከ 1-5 ባለው መለኪያ መሰረት ምን ያህል ይስማማሉ

| የ HMIS ያሎትን እውቀት በተመለከተ | አልስማማም  (1) | አልስማማም (2) | ከሁለቱም ያልሆነ (3) | እስማማለሁ (4) | ፍፁም እስማማለሁ (5) |
| --- | --- | --- | --- | --- | --- |
| 1.HMIS ከአገልግሉትና አስተዳደራዊ መዛግብት መረጃዎችን  ይሰበስባል |  |  |  |  |  |
| 2.HMIS ተግባራዊ የሆኑ ፕሮግራሞችን ለመከታተል ጥቆማ ይሰጣል |  |  |  |  |  |
| 3.HMIS ውሳኔዎችን ለመወሰን ይጠቅማል |  |  |  |  |  |
| 4.HMIS ፖሊሲዎችን ለማውጣትና ለአስተዳደራዊ ውሳኔዎች ይጠቅማል |  |  |  |  |  |
| 5.HMIS ለስራ ለክትትልና ምዘና ይጠቅማል |  |  |  |  |  |
| 6.የHMIS መረጃን በቻርቶች፤በግራፍ እና በሰንጠረዥ ማሳየት ይቻላል |  |  |  |  |  |
| 7.HMISየጤናመረጃስርዓትአካል ነው |  |  |  |  |  |

| በጤና ጣቢያዎ ተቆጣጣሪዎች(የበላይ አለቆች) | አልስማማም  (1) | አልስማማም (2) | ከሁለቱም ያልሆነ (3) | እስማማለሁ (4) | ፍፁም እስማማለሁ (5) |
| --- | --- | --- | --- | --- | --- |
| 1. ከሚመለከታቸው ግለሰቦች ምላሽ ይጠይቃሉ |  |  |  |  |  |
| 2. በወርሀዊ ሪፖርቶች በመረጃ ጥራት ያተኩራሉ |  |  |  |  |  |
| 3. ግጭቶችን ለመፍታት በግልፅ ይወያያሉ |  |  |  |  |  |
| 4. ከሚመለከታቸው ማህበረሰቦች ምላሽ ይጠይቃሉ |  |  |  |  |  |
| 5.የ HMIS መረጃዎችን ግቦችን ለማቀመጥና ለቁጥጥር ይጠቀሙባቸዋል |  |  |  |  |  |
| 6. የመረጃዎችን ጥራት በየጊዜው ያረጋግጣሉ |  |  |  |  |  |
| 7. በሪፖርት አመካይነት ለሰራተኞቻቸው በመረጃ ላይ የተመሰረተ ግብረ መልስ በየጊዜው ይሰጣሉ |  |  |  |  |  |
| 8. የመረጃዎችን ትክክለኛነት በየጊዜው ሪፖርት ያደርጋሉ |  |  |  |  |  |
| 9 ከስራቸው ያሉትን ሰራተኞች ሪፖርቶችን ጨምረው(የውሸት ሪፖርት) እንዲያቀርቡ ያበረታታሉ |  |  |  |  |  |

| የጤና ጣቢያው የስራ አመራር | (1) | Disagree (2) | Neutral (3) | Agree (4) | Strongly Agree (5) |
| --- | --- | --- | --- | --- | --- |
| 1. ለቀን ተቀን የጤና ጣቢያው የስራ አመራር የ HMIS መረጃዎችን ይጠቀማሉ |  |  |  |  |  |
| 2. ያስቀመጡትን ግቦች ለመቆጣጠር መረጃዎችን በግልፅ ያሳያሉ |  |  |  |  |  |
| 3. የችግሮችን መነሻ ለማወቅ የሚያስችሉ መረጃዎችን እንዲሰበሰብ ያበረታታሉ |  |  |  |  |  |
| 4. የችግሮችን መፍትሄ ለመምረጥ የሚያስችሉ መለኪያ ነጥቦችን ማስቀመጥ ይችላሉ |  |  |  |  |  |
| 5. ከተቀመጡት መፍትሄዎች ሊገኙ የሚችሉትን ውጤቶች ይለያሉ |  |  |  |  |  |
| 6. የታቀዱት ግቦች(ውጤቶች) መሳካታቸውን መገምገም ይችላሉ |  |  |  |  |  |

| በጤና ጣቢያዎ ሰራተኞች | አልስማማም  (1) | አልስማማም (2) | ከሁለቱም ያልሆነ (3) | እስማማለሁ (4) | ፍፁም እስማማለሁ (5) |
| --- | --- | --- | --- | --- | --- |
| 1. የሰሩትን ስራ ሁልጊዜ ይመዘግባሉ |  |  |  |  |  |
| 2.የተጠቃሚውን ሀዝብ ጤና ለማረጋገጥ ቁርጠኞች ናቸው |  |  |  |  |  |
| 4.ተገቢና ሊሰሩ የሚችሉ ግቦችን አስቀምጠው ይሰራሉ |  |  |  |  |  |
| 5.የተቀመጡት ግቦች ሳይሳኩ ሲቀሩ የጥፋተኝነት ስሜት ይሰማቸዋል |  |  |  |  |  |
| 6. ለተሰሩት ጥሩ ስራዎች ይሸለማሉ |  |  |  |  |  |

| 7. ውሳኔዎችን መወሰን እንዲችሉ ተፈቀዶላቸዋል |  |  |  |  |  |
| --- | --- | --- | --- | --- | --- |
| 8. ከበላዮቻቸው ሆነ ከስራ ባልደረቦቻቸው ለሚመጡ ማናቸውም በመረጃ ያልተደገፉ ውሳኔዎችና ፍላጎቶች መቃወም ይችላሉ |  |  |  |  |  |
| 9. ለሚከሰቱት ደካማ የስራ አፈፃፀሞች ተጠያቂ ናቸው |  |  |  |  |  |
| 10. ህብረተሰብን ለማስተማርና ለማነቃቃት የHMIS መረጃዎችን ይጠቀማሉ |  |  |  |  |  |
| 11. ስህተቶችን በማረም የማስተካከያ እርምጃዎችን ይወስዳሉ |  |  |  |  |  |

| የግል አመለካከት | (1) | (2) | (3) | (4) | (5) |
| --- | --- | --- | --- | --- | --- |
| 1. ለውሳኔ የማይጠቅም መረጃ መሰብሰብ ይደብረኛል |  |  |  |  |  |
| 2. መረጃን መሰብሰብ ደስ አይለኝም |  |  |  |  |  |
| 3. መረጃን መሰብሰብ ለኔ ትርጉም ያለው ስራ ነው |  |  |  |  |  |
| 4. መረጃን ስሰበስብ የአንድን ተቋም የስራ አፈፃፀም ለመከታተል መረጃው ጠቃሚ እንደሆነ ይሰማኛል፡፡ |  |  |  |  |  |
| 5. መረጃን ስሰበስብ በግዳጅ እየሰራሁ እንዳለ ይሰማኛል፡፡ |  |  |  |  |  |
| 6. መረጃን መሰብሰብ በአለቆችና ሰራተኞቻቸው የተወደደ ነው፡፡ |  |  |  |  |  |

**ከፍል ሶስት ;-የራስን ብቃት መመዘኛ**

የሚከተለው መጠይቅ እርስዎ የጤና ነክ መረጃዎችን በብቃት በመጠቀም ረገድ ያሎትን በራስ መተማመን ለመመዘን ይረዳል፡፡ከፍተኛ በራስ መተማመን አንድን ስራ በብቃት የመከወን ችሎታን ሲያሳይ ዝቅተኛ በራስ መተማመን ደግሞለመሻሻልና ለስልጠና እድል ይከፍታል፡፡ስለዚህ ከHMIS ጋር የተያያዙ ስራዎችን ለመከወን ያልዎትን በራስ መተማመን መወቅ እንፈልጋለን፡፡እባክዎትን በግልፅነትና በሀቀኝነት በራስ መተማመንዎን ይለኩልን፡፡

በራስ መተማመንዎን ለእያንዳንዱ ከታች ለተዘረዘሩት የጤና ነክ መረጃን የተመለከቱ ሁኔታዎች(መገለጫ) በሚከተለው መለኪያ በመቶኛ ይለኩልን፡፡

0 10 20 30 40 50 60 70 80 90 100

| መገለጫ | 0 | 10 | 20 | 30 | 40 | 50 | 60 | 70 | 80 | 90 | 100 |
| --- | --- | --- | --- | --- | --- | --- | --- | --- | --- | --- | --- |
| 1.የመረጃን ትክክለኛነት ማረጋገጥ እችላለሁ |  |  |  |  |  |  |  |  |  |  |  |
| 2. መቶኛዎችን ማስላት እችላለሁ |  |  |  |  |  |  |  |  |  |  |  |
| 3. መረጃዎችን በወራትና አመታት ማስቀመጥ እችላለሁ |  |  |  |  |  |  |  |  |  |  |  |
| 4. ከባር ግራፎች የአከሳሰት ሂደቶችን መገለፅ እችላለሁ |  |  |  |  |  |  |  |  |  |  |  |
| 5.ግኝቶችና ውጤቶቻቸውን መግለፅእችላለሁ |  |  |  |  |  |  |  |  |  |  |  |
| 6.መረጃን ክፍተቶችን ለመለየትና ግቦችን ለማስቀመጥ መጠቀም እችላለሁ |  |  |  |  |  |  |  |  |  |  |  |
| 7. መረጃን የተለያዩ ውሳኔዎችን ለመወሰንና ምላሽ ለመስጠት መጠቀም እችላለሁ |  |  |  |  |  |  |  |  |  |  |  |

**ስለ ትብብርዎ እናመሰግናለን!**
