# Supplementary material for: Data quality assessment and associated factors in the health management information system among health centers of Southern Ethiopia
Source: PLoS One. 2021 Oct 27;16(10):e0255949. doi: 10.1371/journal.pone.0255949 (PMC8550403; doi:10.1371/journal.pone.0255949)
Supplement: Attachment — Submitted filename: comments recommandations.pdf [file pone.0255949.s004.pdf]

DEAR CORRESPONDING AUTHOR/CO-AUTHORS, PLEASE READ BELOW MESSAGE CAREFULLY.

1. Please, rephrase the title. Instead of “Assessment of quality of data” it’s better to adopt the expression “Data quality assessment and associated factors in the Health Management Information System among Health of Hadiya Zone, Southern Ethiopia".
2. The Abreviations must be writing full, at least once, before putting them as abreviations (e.g. SNNPR/EC/HIV/AIDS/TB/ANC/LQAS/RDQA.)
3. Please, erase all extra spaces between words. Ans warning for ponctuations, too. The comma is important, as well as others punctuations (e.g. Data quality was assessed using accuracy, completeness and timeliness)
4. Introduction must be divided into 3 paragraphs : 1. introduction 2. significance of the study and 3. aim of the study.
5. For the results given in the article, the comma in English is not used in maths. (E.g. 23.52 and not 23, 52%)
6. Please insert other references to support your ideas (In introduction for example: (e.g. Therefore the health sector transformation plan (HSTP) considered a need for information revolution as one of the four transformation plan agendas which involves advancement on the methods starting from data collection to the use of information for decision). In this sentence, there are not references.
7. Please, review your English and avoid repeating the expression "In this zone" each time (E.g. …and after the scale up the reformed HMIS was implemented in 2011 ~~in this zone~~. Also (E.g. However, limited researches are done that can show specifically on the level of HMIS data quality and factors affecting data quality ~~in the region~~).
8. Methods : Please, correct the way of writing dates.
9. Methods: Authors can opt for a map, instead of a text for geographic localization or rephrase the paragraph.
10. Please, reformulate this sentence: (The study utilized Facility based …..health workers involved in RHIS activities).
11. The steps in methods are to be restructured. You can combinate between some points. In addition, there are many repetitions.
12. This forms (►) are not accepted in scientific articles. Another format will be more professional.
13. For the results part, please reclassify the axes, and summarize them so that the information will be clearer.
14. There are empty boxes in your table 5.
15. Tables are also to be combined.
16. The form of references is not that requested by PLOSONE revue.
