# Supplementary material for: Data quality assessment and associated factors in the health management information system among health centers of Southern Ethiopia
Source: PLoS One. 2021 Oct 27;16(10):e0255949. doi: 10.1371/journal.pone.0255949 (PMC8550403; doi:10.1371/journal.pone.0255949)
Supplement: Attachment — Submitted filename: manuscript on data quality with the remarks.docx [file pone.0255949.s003.docx]

**Assessment of Quality of data and associated factors in the Health Management Information System among Health Centers of Hadiya Zone, Southern Ethiopia**

Mastewal Solomon*^1^ Mesfin Addise^2^	Berhan Tassew^2^	Bahailu Balcha^3^

^1^Hadiya zone health department, Shone town administration Health Office

^2^Addis Ababa University, College of Health sciences and Medicine, School of public Health, Addis Ababa, Ethiopia

^3^Wolaita Sodo University, College of Health sciences and Medicine , School of Public Health, Wolaita Sodo, Ethiopia

Corresponding authors: Mastewal Solomon

Email addresses :

[massfiker2012@gmail.com](mailto:massfiker2012@gmail.com) [shewit91@yahoo.com](mailto:shewit91@yahoo.com) [tassewberhan@gmail.com](mailto:tassewberhan@gmail.com) [behailubalcha2@gmail.com](mailto:behailubalcha2@gmail.com)

**Abstract**

**Background:** A well designed Health management information system is necessary for improving health service effectiveness and efficiency. It helps to produce quality information and conduct evidence based monitoring, adjusting policy implementation and resource use. However, evidences show that data quality is poor and is not utilized for program decisions in Ethiopia especially at lower levels of the health care and it remains as a major challenge.

**Objective:** To assess the level of quality of data and associated factors in the routine health management information system among Health Centers of Hadiya Zone in 2018.

**Method:** Facility based cross sectional study design was employed using quantitative method. A total of 18 health centers and 302 health professionals were selected using simple random sampling procedure. Data was collected by health professionals who were experienced and had training on HMIS tasks after the tools were pretested. Data quality was assessed using accuracy completeness and timeliness dimensions. Seven indicators from national priority area were selected to assess data accuracy and monthly reports were used to assess completeness and timeliness. Statistical software SPSS version 20 for descriptive statistics and binary logistic regression was used for quantitative data analysis to identify candidate variable.

**Result:** A total of 291 respondents were participated in the study with response rate of 96%. Overall average data quality was 82.5%. Accuracy, completeness and timeliness dimensions were 76 %, 83.3 and 88.4 respectively which was lower than the national target. Over reporting was observed in all facilities and on all indicators. About 52.2% respondents were trained on HMIS, 62.5% had supervisory visits as per standard and only 55.3% got written feedback. Only 11% of facilities assigned health information technicians. Level of confidence [AOR=1.75, 95% CI (0.99, 3.11)], filling registration or tally completely [AOR= 3.4**, 95% CI (1.3, 8.7)]**, data quality check, supervision AOR=1.7 95% CI (0.92, 2.63) and training [AOR=1.89 95% CI (1.03, 3.45)] were significantly associated with data quality.

**Conclusion:** This study found that the overall data quality was lower than the national target. Over reporting of all indicators were observed in all facilities. It needs major improvement on supervision quality, training status to increase confidence of individuals to do HMIS activities.

# Introduction

Health management information system (HMIS) is one of the six building blocks of health system that integrate data collection, processing, reporting, and use of the information. It is necessary for improving health service effectiveness and efficiency through better management at all levels of health services. It is also the foundation of decision-making across all health system building blocks, and is essential for health system policy development and implementation, governance and regulation, health research, human resources development, health education and training, service delivery and financing (1,2).

Globally, the restructuring of health information systems has been an important trend since its declaration in Alma-Ata conference of primary health care as an essential health care strategy in 1978 (1,3).

Developing countries also launched reforms to improve and expand health information systems as a component of health system reform (4).

The Ethiopian HMIS has been implemented since 2008 and provide core indicators used to improve the provision of health services, and ultimately, to improve health status of the population. It is a major source of information for monitoring and adjusting policy implementation and resource use. (5).

The routine health information system implemented since 2009 and after the scale up the reformed HMIS was implemented in 2011 in the zone. (6).

The need for organized, accessible, timely, and accurate data for health decision making has become a growing concern. In response to this, the Ethiopian FMOH has undertaken an extensive reform and redesign of the national HMIS. The reform has taken major steps to respond to the deficiency of routine health data that limited the quality of care, planning, and management systems, as well as decision making by managers at all levels in the health care system.(7).

Therefore the health sector transformation plan (HSTP) considered a need for information revolution as one of the four transformation agendas which involves advancement on the methods starting from data collection to the use of information for decision. The focus of information revolution is not only on the method of advancement but also on the changes of culture and attitude toward information use. Improving health system efficiency and effectiveness through the guiding principles of standardizing, recording and reporting forms, integration, simplification, human resource development and ICT applications..(7,8).

Data that are accurate, complete and delivered on time to users is an important aspect in healthcare planning, management and decision making but quality of data is frequently assessed as a component of the effectiveness or performance of the HIS; however data quality assessment is hidden within these scopes. This may lead to ignorance of data management and thereby the unawareness of data quality problem (9).

In Ethiopia, data quality and reliability issues are not well guiding program decisions in all aspects. Poor data quality at the lower administrative level or peripheral levels of woreda and health facilities, which are the source for majority of data used for decision making in the health sector remains a challenge as reported in 2016 annual reports of health sector transformation plan..(10).

Lack of accuracy, timeliness and completeness of HIS reporting remains a weakness, and such delays contribute to the challenge to use data as the basis for informed decision-making in health care planning and management.(11). According to the assessment conducted on HMIS data quality and information use showed content completeness, reporting timeliness and accuracy were 39% ,73% and 76% respectively (12).

Existing evidences shows in Ethiopia including SNNPR low level of data quality was reported as a gap which was below the national standard. Data accuracy level for health centers was 36.22% which was much lower than the national target. This is due to many factors like lack of training, lack of decision based on supervision, lack of feedback, data quality assurances are done less frequently, limited skills of the health professionals. (6,7,11).

Even though, as reported on the 2008 EC annual HSTP performance report of SNNPR, improvements have been seen in HMIS performance in the region, there is still a challenge in data quality especially on indicators related with HIV/AIDS, TB and ANC. However, limited researches are done that can show specifically on the level of HMIS data quality and factors affecting data quality in the region. (13).

The annual report of Hadiya Zone in 2009 EC also showed there was a gap in completeness and timeliness of reports. The LQAS assessment result also showed discrepancy of the reports for accuracy of data, over and under reporting of results and doesn’t much expected level of RDQA proportion (0.90-1.10) (14). This indicates that the need to assess the level of data quality and factors associated with data quality in the area to improve the quality and its use in decision making in the zone.

# Methods

This study was conducted from March 15/2018 – April 15/2018 in Hadiya Zone, which is found in the Southern, Nations, Nationalities and Peoples` Regional State of Ethiopia, comprises of 10 woredas, 2 town administration and 333 kebeles (305 rural kebeles and 28 urban kebeles). Its capital is Hosanna town, which is located 205 KM away from Addis Ababa. It is bordered by Gurage Zone in the North, Kembata Zone & Halaba special woreda in the South, Silte Zone in the East and Yem Special Woreda & Omo River in the west. The zone has one general hospital, 2 primary hospitals, 61 health centers and 309 health posts. The zone has 2716 health professionals from different disciplines . (15).

## The study utilized Facility based cross sectional study design. The Study Population included Randomly selected functional health centers of Hadiya zone and Randomly selected health workers involved in RHIS activities.

## Sample size determination

For Accuracy dimensions

Samples of 18 Health centers were selected to assess data quality. Based on the national HMIS information use and data quality manual, seven to nine data elements from each health center is satisfactory to assess data accuracy(16). Data elements were selected randomly from top priority indicators at national level. Therefore, seven data elements from the 18 selected health centers were verified. 2 month documents were reviewed to see consistence of selected data elements of by random selection of the months September and November. The accuracy of data elements was determined by Accuracy Ratio (recounted data from the source document or registrations over reported data to the next level) for the respective data element. Lower than 0.90 or 90% accuracy ratio indicates over-reporting and higher than 1.10 or 110% accuracy ratio indicates under-reporting.

Seven data elements, Antenatal care fourth visit, institutional deliveries, Pentavalent third doses, PMTCT coverage, Tuberculosis cure rate, confirmed malaria cases, and Contraceptive accepters rate were selected.

For Completeness and Timeliness

Content completeness was assessed by proportion of filled data elements of reporting formats pertaining to selected months. A tolerance level of 90 percent was used in grading health centers, which meant that each health center expected to complete at least 90 percent of data elements on report formats. All data elements of two months HMIS reports were reviewed to assess content completeness of reports.

Timeliness also assessed by proportion of facilities with number of reports delivered up to deadline come for the selected two months. A tolerance of 90% was used in grading health centers.

**Sample size for the respondents self-administered Questionnaires**

Sample size was calculated using single population proportion formula based on the following assumption ,75% of peoples capable of performing HIS tasks in Eastern Ethiopia (9), desired degree of precision was 5% , 95% of confidence interval. These results the sample size of 288 and using a contingency of 5 % for non-respondents the final sample size will be 302.

**Sampling procedures**

Health professionals for self administered questionnaire were selected by using simple random sampling technique.

WHO recommended for assessment of health facilities by considering the available funds and human resources, selecting 10%-50% facilities to have representative sample. Among the total 61 health centers in the zone 30% of health centers were selected based on the suggestion (17) . A total of 18 health centers were selected by using simple random sampling technique. The calculated sample size for respondents Self administered questionnaire was proportionally allocated to each health center, then health professionals were selected randomly who are involved in HMIS activities starting from daily register of the source document to the final report were included.

Health centers that are functional for more than one year were included but Health professionals from new health centers for less than one year and Health workers who had less than six month experience were excluded.

**Data collection instrument and procedures**

Data collection tools were adapted from the PRISM assessment tools version 3.1 and HMIS user’s guideline. The tool is prepared to fit with local context and it mainly contains questions to assess accuracy, completeness and timeliness HMIS data. Self-administered structured questionnaire containing back ground information of the respondents organizational , behavioural and technical determinants of data quality in health centers (16,18).

The tool was pretested prior to actual data collection period on 5% of the sampled health professionals and they weren’t included in the actual data collection

The collected data were checked for the completeness and coded before entry and entered to EPI info version 7 then exported to SPSS version 20 for processing and analysis through descriptive statistics. Incomplete, inconsistent and invalid data were refined properly to get maximum quality of data before, during and after data entry. Percentage, Frequency distribution tables and figures were used to describe the study variable for assessment of HMIS.

Binary logistic regression was used to identify the association between problems in data quality and the factors. Bivariable analysis was conducted and variables with p <0.25 selected as candidate variables for multivariate analysis. Finally variables with p<0.05, during multivariable analysis was considered as significant.

The overall data quality was calculated by taking the sum of completeness, timeliness and accuracy scores.

The dependent variable were HMIS data quality while the following factors were included in the model as independent variables: Socio-demographic Factors**:** Age, Sex, Education level, Position of respondents, Work experience :Technical factors;-Complexity of the reporting formats and procedures, Availability of Computer software’s (data base), Standard set of indicators with definition.: Individual behavioural factors:- Knowledge of content of HMIS form ,Confidence levels for HIS Tasks, Data quality checking skill, Motivation, incentives : Organizational factors;- Management support for HMIS, Training, Supervision, Regular feedback

## Data quality management

To ensure the quality of data the following activities were done: adapting questionnaires from Standard tools, then translated in to Amharic. Training was given to data collectors on sampling procedures, techniques of interview and data collection process and supervised by the principal investigator. Pre testing of questionnaire was undertaken to check the understandability by taking 5% of sample from other health centers which are not included in the actual data collection. Inconsistent and incomplete data were managed accordingly before data entry in computer software’s.

.

**Variables measurement**

- **Data accuracy;-**was measured by calculating the number from source document over the number from report submitted to the next level. Based on 10% tolerance for data accuracy was classified as follows;-

Over reporting (<0.90 or 90%),

Acceptable limit (0.90-1.10 or 90%-110%)

Under reporting (>1.10 or 110%)

- **Content completeness** was measured by the number of cells of report form which are left blank without indicating “zero”. If greater than or equal to 90% of cells of the report filled was considered as complete.
- **Report timeliness** was measured by the number of reports delivered up to deadline for facility head over the number of reports expected to come
- **Level of Knowledge:** A health professional said to be knowledgeable if they responds knowledge questions above respondent mean score**.**
- **Confidence level or Self-efficacy;-**was measured in a scale of 0-100 that means from no confidence (zero) to full confidence (100) to perform HMIS tasks.

**Ethics approval and consent to participate**

The ethical approval for this study was obtained from the research ethical committee of school of public health, Addis Ababa University; permission letter was written for AARHB, Hadiya zone health office, woreda health office and health centers. Then informed written consent was obtained from the participants, after the necessary explanation about the purpose, procedures, benefits, risks of the study is explained and also their right on decision of participating in the study. After getting informed consent from the respondents the right of the respondents to refuse answer for few of all of the questions was respected.

**RESULT**

Characteristics of respondents

A total of 291 respondents were participated in study with response rate of 96%. Eleven health centers head (3.8%), 137 department heads (47%), 15 HMIS focals (5.2%) and 128 service providers (44%) were participated in the study. Most of the respondent’s age was within the range of 21-30(71.1%). Among the respondents 62.5% were male. Regarding distribution of level of education 190 (65.3%) were level four diploma holders and 101 (34.7%) bachelor degree holders. About 56.7% the respondents were nurses with the maximum experience of 10 years and average experiences of 5 years.

Table 1: Socio demographic characteristics of respondents in health centers of Hadiya zone, Southern Ethiopia, 2018 [n=291].

| Variables | Category | Frequency | Percent (%) |
| --- | --- | --- | --- |
| Sex of respondents | Male | 182 | 62.5 |
|  | Female | 109 | 37.5 |
| Age of respondents | 20-24 | 35 | 12.1 |
|  | 25-29 | 139 | 47.9 |
|  | 30-34 | 71 | 24.4 |
|  | 35-39 | 25 | 8.6 |
|  | 40-44 | 21 | 7.2 |
| Educational status | Diploma | 190 | 65.3 |
|  | Bachelor degree | 101 | 34.7 |
| Years of experience | 1-5 | 182 | 62.5 |
|  | 6-10 | 109 | 37.5 |
| Position of respondents | head of health centers | 11 | 3.8 |
|  | department heads | 137 | 47 |
|  | HMIS focals | 15 | 5.2 |
|  | Service providers | 128 | 44 |
|  | Midwife | 45 | 15.5 |
|  | Health officer | 46 | 15.8 |
|  | Laboratory technician | 20 | 6.9 |
|  | Pharmacy | 13 | 4.5 |
|  | HIT | 2 | 0.7 |

## General structure and capability of HMIS

All health centers assigned HMIS focal persons who are responsible for reviewing and aggregating numbers prior to submission to the next level. About 11 health centers assigned HMIS focals who are engaged on other responsibility like service provision. Only 11% of facilities assigned HIT professionals.

Based on the finding only 4 health centers were using functional computer software and all have Rules to prevent unauthorized changes to data (password). All 18 health centers were established performance monitoring team.

Table 2: General structure and capability of HMIS in health centers of Hadiya Zone, southern, Ethiopia 2018.

| **Variables** | **Expected No- of items** | **Observed No- of items** | **%** |
| --- | --- | --- | --- |
| HMIS focal person | 18 | 18 | 100 |
| have written job descriptions | **18** | 0 | 0 |
| Electronic data base (computer software) | 18 | 5 | 28 |
| currently functional computer software | 18 | 4 | 22 |
| Rules to prevent unauthorized changes to data | 18 | 4 | 22 |
| Establish performance monitoring team | 18 | 18 | 100 |

## Record keeping

All health centers kept copies of reports. The count for one year period of copies of reports shows that the monthly report kept ranges from 10-12. From all health centers assessed 96% kept copy of monthly reports that are sent to the next level.

## Accuracy of data

A total of 18 health centers were studied for data quality by accuracy, completeness and timeliness dimensions. Seven data items or indicators were assessed for data accuracy. Service delivery reports and registration books were checked for the month September and November by random selection of the months. Seven indicators verified were Antenatal care fourth visit(ANC 4), Contraceptive acceptance rate (CAR), Institutional delivery, Pentavalent third doses (Penta 3), PMTCT, TB cure rate and confirmed malaria cases from top priority indicators at national level.

From 18 facilities observed 44% of facilities were within acceptable level of accuracy. Data were over reported in all facilities. ANC4 and PMTCT data was over reported by 14 health centers (78%). About 11% health centers under reported TB cure rate and confirmed malaria cases. 14 health centers over reported. Only three out of seven (42.8%) indicators were within 10% acceptable level. About 19% of ANC4 data, over reported (>10% tolerance level) followed by 16%, 15% and 14% CAR, Penta3 and PMTCT data were over reported (>10%). The overall accuracy of data was 76%.

## Completeness of data

Content completeness was assessed by checking two months service delivery report whether the required data elements in a report form are filled or data are complete. Overall content completeness was 83.3%.

## Timeliness of data

Timeliness of the HMIS data was assessed by checking whether HMIS data reporting by the health facilities met the predetermined deadline of reporting period received by the facility head. Over all timeliness was 88.42%. About 55.5% facilities found within 90% tolerance level.

Based on the three dimensions of data quality which are accuracy, completeness and timeliness the overall data quality of the health centers was 82.5%.

**HMIS process**

Concerning participation of respondents in HMIS activities among the respondents 87.3% participate in aggregation or compilation of data from registration. More than half the of respondents (57.7%) reported that they conduct data quality check but frequency of conducting data quality varied among respondents that about 51.8% conduct data quality test on monthly basis. Overall 86.9% of the respondents reported that they fill registration or tally sheet completely.

## Technical factors

From total respondents 59.8 % of respondents were reported that they had standard set of indicators including case definitions in their departments. Among the respondents 40.5 % reported that there are skilled staff able to aggregate data and to fill out formats and 77.7% reported that HMIS is user friendly format.

Table 3 Technical factors of HMIS data quality in health centers of Hadiya zone, southern Ethiopia 2018.

| **Technical factors** | **Yes (%)** | **No %** |
| --- | --- | --- |
| Standard set of indicators including case definitions | 174(59.8) | 117(40.2) |
| Skilled staff able to aggregate data and to fill out formats | 118 (40.5) | 173(59.5) |
| Complexity of HMIS formats(user friendly format ) | 226 (77.7) | 65 (22.3) |

## Behavioural factors

Individual behaviour factors were assessed through individual perception (motivation) towards HMIS use, knowledge of respondents regarding HMIS, confidence level of respondents to do HMIS tasks and availability of incentives for HMIS for HMIS activities. About 28% of respondents reported that availability of incentives for HMIS activity which is training opportunity. About 60.8% of respondents had knowledge towards HMIS. About 66% reported on data quality checking skill and average confidence level of respondents was 63%. Average perception (motivation) of individuals towards HMIS use and meaning was 49.1%.

Table 4: summary of behavioural factors of HMIS in health centers of Hadiya zone, southern Ethiopia 2018.

| Behavioural factors | Yes (%) | No (%) |
| --- | --- | --- |
| Incentives | 82 (28) | 209 (72) |
| Knowledge on HMIS | 177(60.8) | 114(39.2) |
| Data quality checking skill | 192(66) | 99 ( 34) |
| Individual perception(motivation) | 143 (49.1) | 148 (50.9) |
| Self efficacy (confidence level) | 183( 63) | 108(37) |

**Self efficacy**

Confidence level to perform HMIS tasks for health professionals were assessed on a scale of 0 to 100. The average score obtained for the seven questions expressed as a percentage. Higher confidence was observed in checking data accuracy and calculating percentages (66%) and lower confidence was observed in explaining findings from bar charts (56%) relatively. The average confidence level to perform HMIS activities of respondents were 63%.

## Organizational factors

Regarding training status, from the total respondents 52.2% reported that they had received training on HMIS activities. Among those 35.1% took in-service training related with HMIS tasks. From total respondents 62.5% of respondents supervised one times in last three months from higher officials regarding data quality. Regarding feedback, 55.3% of respondents received feedback from next higher official’s among those 60.2% get feedback reports monthly. About 60.8% of respondents agreed on extent of management support regarding HMIS activities.

Among the respondents 61.9% of respondents agreed on, their supervisors give emphasis for data in monthly reports and 55% agreed that supervisors provide regular feedback to their staff. About 63.2% the respondents agreed on, their supervisors check data quality regularly. About 44.3% of respondents agreed on their supervisors encourage over reporting of data for underperformed reports

**Multi-variable analysis.**

Variables with p<0.05 taken as predictor of HMIS data quality. Training has shown significant relationship (P<0.05) with data quality **[AOR=1.89, 95% CI (1.03, 3.45)].** Those who were trained 1.89 times more likely to report quality data than who were not trained. Filling registration or formats completely also show significant relationship with data quality **[(AOR=3.4 95% CI (1.3, 8.7)].** Those who fill the registration or formats were 3.4 times more likely report quality data than those who were not fill completely. Self efficacy (perceived level of confidence) has significant relationship with data quality **[AOR=1.75 95% CI (0.99, 3.11)].** Those who have high level of confidence were 1.75 times more likely to report quality data than those who have low confidence level. Supervision has significant relationship with data quality **[AOR= 1.7 95% CI (1.00, 2.95)].** Those supervised health workers were 1.7 times more likely to report quality data compared to who were not supervised. Checking data quality also has significant relationship with data quality **[AOR=1.8 95% CI (0.49, 3.09)]**. Those health workers who conduct data quality check were 1.8 times more likely to report quality data compared to who were not.

Table 5: Multi variable logistic regression result on data quality for health centers of Hadiya Zone southern Ethiopia 2018.

| Variables | | data quality | COR (95% CI) | AOR (95% CI) | P- value |
| --- | --- | --- | --- | --- | --- |
| Knowledge on HMIS | Yes | 177(60.8%) | 1.99(1.18,3.35) | 1.209(0.29,2.72) | 0.84 |
|  | No | 114(39.2%) |  |  |  |
| filling registration or tally completely | Yes | 253(87%) | 4.42(2.2,8.9) | 3.41*(1.3,8.7) | 0.043 |
|  | No | 38(13%) |  |  |  |
| Supervision | Yes | 182(62.5%) | 1.56(0.92,2.63) | 1.71*(1.00,2.95) | 0.037 |
|  | No | 109(37.5%) |  |  |  |
| Training | Yes | 152(52.2%) | 1.59(0.95,2.67) | 1.89*(1.03,3.45) | 0.014 |
|  | No | 139(47.8%) |  |  |  |
| Confidence level | Confident | 183(63%) | 1.71(1.01,2.9) | 1.75*(0.99,3.11) | 0.047 |
|  | Not Confident | 108(37%) |  |  |  |
| Data quality check | Yes | 168(57.7%) | 1.78(1.06,2.9) | 1.8*(0.49,3.09 | 0.032 |
|  | No | 123(42.3%) |  |  |  |
| Complexity of the formats | Yes | 226(77.7%) | 1.69(0.94,3.04) | 0.70(0.32,1.50) | 0.36 |
|  | No | 65(22.3%) |  |  |  |
| Management support | Yes | 177(60.8%) | 1.99(1.18,3.35) | 0.89(0.29,2.71) | 0.84 |
|  | No | 114(39.2%) |  |  |  |
| Availability of procedural manual | Yes | 146(50.2%) | 1.52(0.908,2.54) | 1.41(0.82,2.44) | 0.22 |
|  | No | 145(49.8%) |  |  |  |
| Sense of responsibility | Yes | 175(60.2%) | 2.05(1.22,3.44) | 1.33(0.43,4.13) | 0.62 |
|  | No | 116(39.8%) |  |  |  |
| Standard set of indicator | Yes | 174(59.8%) | 1.87(1.69,4.84) | 2.10(0.77,5.73) | 0.144 |
|  | No | 117(40.2%) |  |  |  |
| Educational status | Diploma | 190 (65.3%) |  |  |  |
|  | Degree | 101(34.7%) | 1.65(0.94,2.90) | 1.52(0.84,2.74) | 0.16 |

* shows predictor variables for information utilization at p<0.05

**Discussion**

Quality of data is a key factor in generating reliable health information that enables monitoring progress and making decisions for continuous improvement(7). However the quality of data in the zone based on accuracy, completeness and timeliness showed 76%, 83.3% and 88.4% respectively. Overall data quality of the zone scored 82.5% which was below the national target 85% (5).

All decision of the health system depends on the availability of timely, accurate, and complete information. However the study found 76% of data accuracy. The finding was comparable with the assessment done in Ethiopia, 76% of data accuracy level reported (12). However According to the baseline assessment done in SNNPR, 36.22% of data accuracy was observed at health centers which was lower than the current study (6). This may be due to the time gap, 7 years between the studies

Out of 18 health centers 8(44%) health centers were in acceptable level of data tolerance. This finding was supported by the study done in India, 63% facilities were not in acceptable limit of data accuracy (19).

Discrepancy of data was observed in all facilities, what is on register and on report formats. Tendencies of over reporting in all indicators and under reporting of some indicators were observed. The finding was similar with an evaluation done in Tigray region (20). This may be due to incompleteness of data, not understanding the definition of cases or data elements, or data may not fall within the reporting period (16).

Data were over reported in all facilities. ANC4 and PMTCT data was over reported by 14 health centers (78%). This is supported by a national assessment done by EPHI. From the indicators assessed over reporting was observed in ANC and FP services. The study showed only 30 percent of ANC data reported was matched with source document but in this study about 88% of ANC4 data was matched. The improvement may be due to the study was nationwide so that including many institutions probably increase inclusion of those facilities with low data quality. Delivery data were over reported about 8% which was similar with EPHI data over reporting >10% (21).

About 11% of health centers under reported TB service data and confirmed malaria cases. PMTCT and ANC data was over reported by 14 health centers. From the indicators assessed, only three out of seven (42.8%) indicators were within 10% acceptable level. About 19% of ANC4 data, over reported (>10% tolerance level) followed by 16%, 15% and 14% CAR, Penta3 and PMTCT data were over reported (>10%). About 39 %of health centers over reported delivery data. This was also comparable with EPHI national assessment where Proportions of public facilities made greater than 10 percent over (20%) of Penta3 data, 88% PMTCT data was the best-matched data among all indicators (21). This may be due to the fact that the indicators are from the top priority indicators at national level and needed to be performed well which might lead the facilities to over report and it may also be due to manual entry of data. According to the new information revolution every facility expected to use electronic HMIS but in the studied facilities only four facilities use functional electronic HMIS software (data base).

Regarding content completeness the result found 83.3% of completeness based on 90% tolerance, which was slightly higher than a study conducted in Ayder referral hospital 78.6% and a systematic review conducted in Ethiopia (12,22). Whereas the result was comparable with a study conducted previously in the same setting on HMIS utilization 82.8%(23).

Another dimension of data quality was timeliness which is measured by, facilities receiving case teams’ reports by the predetermined deadlines. Overall timeliness scored 88.4% based on 90% tolerance of timeliness which was higher result from study done in SNNPR 77% (6,12). The result also revealed better achievement when compared to study conducted previously in the same setting, only 59.6% reports submitted on recommended time period (14).

Content completeness and timeliness dimensions showed less achievement from a study done in Tigray region and Rwanda where 100 percent facilities met 90% data tolerance (20,24). Possible reasons may be due to lack of knowledge of respondents about the implications of an incomplete data on a report formats and to send reports on timely manner among the health workers and it may also be less emphasis was given for data quality during supervision.

Odds of data quality on those health workers who were filling the source document (registration or tally), higher than those who were not filled [AOR **3.4, 95% CI (1.3, 8.7**)]. Similar finding was found on a studies done in Jimma and Bahir Dar town (25,26). This may be due to non understandability (complexity) of the tools/formats, using of untrained workers or shortage of training supports on the forms and registers. So that it is difficult to register all relevant information in correct manner and retrieval of these data will be trouble full.

Concerning supervision, regular Supportive supervision with feedback is a key in addressing quality issues by helping to improve overall performance of HMIS especially for better achievement of data quality (27). More than half (62.5%), health centers participated in this study supervised by their respective higher level as per standard in the last two quarters. The result was supported by studies conducted previously in Dire Dawa and SNNPR (6,11). Even though the result was comparable with other studies conducted earlier, about 37.2% health centers were not supervised regularly. One of the most important mechanisms to improve quality of data is regular supervision. Lack of regular systems on supportive supervision affects the importance and quality of data collection. Therefore without regular and program specific supportive supervision it is difficult to achieve information transformation.

Regarding training, continuous training on HIS activity is important to create awareness and to have trained staff and skilled human resources that are confident and motivated to perform HIS tasks (25). This study found about 52% of health workers trained regarding HMIS activities. This finding was comparable with other studies done in Dire Dawa 52.7% and South Africa 58% were not trained related with HMIS activities (26,28). All health workers who participate in the collection at various sections of healthcare, need continuous capacity building to conduct quality review of RHIS at every stage for in-depth understanding of the stages where quality of data can occur (28,27). In this study all focal persons and department heads trained regarding HMIS activities but others, service providers who were not trained were involved in the process of HMIS.

Odds of health information data quality among Health workers those who were confident enough to perform HMIS activities were higher than those who were not confident [AOR=1.75, 95% CI (0.99, 3.11)]. The result was supported by studies conducted in SNNPR and South Africa (6,26). This factor also suggested by WHO measure evaluation as one determinant of data quality (18). This may be due to complexity of the formats/tools. If data collection forms are complex to fill in, it affects confidence levels and motivation of data collector(18)**.**

Concerning data quality check, good data management require data quality check at all stages. The checking of data quality is the responsibility of all health workers participating in the data management (30).In this study about 57.7% of health workers check data quality with a frequency of 51.8% on monthly basis. This is supported by different literatures in done by WHO measure evaluation and a study done in Kenya. From a study done in Kenya about 63% of respondents check data quality but the frequency of carrying out the checks was varying from one respondent to another with majority indicating every quarterly 22%(18,23,29).

# Conclusion and recommendations

## Conclusion

Data quality for the three dimensions was 82.5% which is lower than the national target 85% for data accuracy. Over reporting of data was observed at all facilities. About 39 % of health centers over reported delivery data. About 9% data of ANC4 over reported (>10% tolerance level) followed by 6%, 5% and 4% CAR, Penta3 and PMTCT data were over reported (>10%). Decisions made using inaccurate, incomplete and reported not on timely manner can affect the health system performance. It was observed that there were inadequacy of supervision, training, HIT professionals, written feedback and procedural manuals. The major factors that affect quality of data were, filling registration or tally completely, training, supervision, data quality check and confidence level. Computerized HMIS data base should be distributed for those who are not using; as it will help to improve data accuracy, timeliness of report and reduce the burden of data collectors

**Acknowledgments**

Our gratitude goes to supervisors, data collectors, respondents , Hadiya zone health department.

**Authors’ contributions**

Mastewal Solomon : Involved from the conception and design, acquisition of data, analysis and interpretation, Dr Mesfin Addise : Involved from the inception to design, acquisition of data, analysis and interpretation and drafting the manuscript, Berhan Tassew : Involved in the analysis & interpretation of data, Bahailu Balcha : Involved in interpretation and manuscript preparation. Finally all authors read and approved the final manuscript.

**Competing interests**

The authors declare that they have no competing interests

**Funding**

This study has been sponsored by Addis Ababa University

The data will be available on request

# Reference

1.	Theo L, Sauerborn R and, Bodart C. Design and implementation of health information systems. WHO. 2000;

2. WHO. Every body ’ s business strengthening health systems to improve health outcomes W HO’s frame work for action. 2000;

3.	Chet N Chaulagai, Christon M Moyo, Jaap Koot, Humphrey B Moyo, Thokozani C Sambakunsi FMK and PDN. Design and implementation of a health management information system in Malawi issues , innovations and results. Oxford University Press. 2005;volume 9.

4.	Vital wave consulting. Health Information Systems in Developing Countries. 2009.

5.	FMOH. Health Sector Transformation Plan. 2015;

6.	Hiwot B, Tariq A, Kassahun H. Assessment of Health Management Information System ( HMIS ) Performance in SNNPR , Ethiopia. 2011;

7.	FMOH. Information Revolution Roadmap. 2016;(April).

8.	FMoH. Health Sector Transformation Plan. 2015.

9.	Chen H, David H, Ning W, Ping Y. A Review of Data Quality Assessment Methods for Public Health Information Systems. International Journal of Environmental Research and Public Health. 2014;5170–207.

10.	FMOH. Health sector transformation plan-I annual performance report (ARM). 2016.

11.	Kidist T, Kidane T, Mirutse G, Wondwossen T. Level of data quality from Health Management Information Systems in a resources limited setting and its associated factors , eastern Ethiopia. South African Journal of Information Management. 2004;1–8.

12.	Mesfin G, Hajira M, Habtamu T, Dereje M, Nafo TF. Data quality and information use : A systematic review to improve evidence ,. :53–60.

13.	SNNPR. health sector transformation plan annual performance report. 2016.

14.	Hadiya zone health department. Hadiya Zone Annual Service delivery Aggregate. 2017.

15.	Development SB of F and E. SNNPR Annul Statstical Abstract 2015/2016. 2016.

16.	FMOH. HMIS Information Use Guide. 2013.

17.	Sambo LG, Chatora RR, Simone G. Tools for Assessing the Operationality of District Health Systems. Brazzaville; 2003.

18.	Hiwot B, Theo L. Inventory of PRISM Framework and Tools: Application of PRISM Tools and Interventions for Strengthening Routine Health Information System Performance. 2013.

19. Dr Harikumar S. Evaluation of Health Management Information Systems. 2012.

20. Ataklti W, Kidane T, Teklit G. Process Evaluation of Health Management Information System Implementation Status in Public Health Facilities of ... Process Evaluation of Health Management Information System Implementation Status in Public Health Facilities. 2017;(January).

21.	Mebrahtu M. Assessment of Health Management Information System in Harari Regional State. 2010.

22.	Kidane T, Ejigu G, Girma T. Assessment of health management information system implementation in Ayder referral hospital , Mekelle , Ethiopia. 2014;3(4):34–9.

23.	Abera E, Daniel K, Letta T, Tsegaw D. Utilization of Health Management Information System and Associated Factors in Hadiya Zone Health Centers , Southern. 2016;1(2).

24.	Innocent K, Robert A, Simon P, Philip G. Quality and Use of Routine Healthcare Data in Selected Districts of Eastern Province of Quality and Use of Routine Healthcare Data in Selected Districts of Eastern Province of Rwanda. 2016;

25.	Sultan A. Utilization of health information system at district level in Jimma zone Oromia regional state , South West Ethiopia.

26.	Helen Tesema. Assessment of the health management information system (HMIS) implementation status in public health facilities and inisituitions in Amhara region the case of Bahirdar city administration. 2011.

27.	FMoH. Health Management Information System (HMIS) / Monitoring and Evaluation (M&E). 2008;

28.	Samuel, K C and GWO-O. Process factors influencing data quality of routine health management information system : Case of Uasin Gishu County referral Hospital , Kenya. International Research Journal of Public and Environmental Health. 2016;3:132–9.

29.	Samuel, Kiprono Cheburet, and George, Odhiambo OW. Organizational factors affecting data quality of routine health management information system quality : Case of Uasin Gishu County Referral Hospital , Kenya. International Research Journal of Public and Environmental Health. 2016;3:201–8.
