# Supplementary material for: Data quality assessment and associated factors in the health management information system among health centers of Southern Ethiopia
Source: PLoS One. 2021 Oct 27;16(10):e0255949. doi: 10.1371/journal.pone.0255949 (PMC8550403; doi:10.1371/journal.pone.0255949)
Supplement: Attachment — Submitted filename: Response to reviewers.docx [file pone.0255949.s005.docx]

**Response to reviewers**

First of all, we would like to acknowledge the academic editor for giving us adequate time to revise and address all the concerns of the reviewers and journal requirements. Following, we the authors of this manuscript have been working extensively since we have been notified with the academic editor and expert reviewer’s report of the manuscript giving a due attention for all the concerns raised by the academic editor and expert reviewers to be well addressed. Thank you so much!

A. **Point by point response letter to academic editor**
1. We have checked again our manuscript for fulfillment of PLOSONEs style requirements including the naming of files and it has been written accordingly. Thank you!!
2. Regarding the language usage, grammar and spelling errors, we have re-written each and every statement throughout the manuscript correcting all the grammatical, spelling and punctuation errors consulting English language professionals. Thank you!
3. According to the academic editor’s recommendation to include the participating health facilities in the methods or as the supplementary file, the participating health institutions are included as supplementary file. Thank you!
4. As per the request of the academic editor we have included all the survey questionnaire (in the original language and the English version) as supporting information. Thank you. Thank you in advance!!!
5. Regarding the data availability all the data used are included in the manuscript. Thank you!
6. According to the academic editor’s recommendation regarding publicity of the funding source, we have removed the statement from the acknowledgment section in the current version Thank you in advance!!!
7. Regarding inclusion of the author Berhan Tessaw in the online submission, we have included (via edit submission) online and included one additional author (Amene Abebe) who was missed in the first submission. Thank you!
8. The comment regarding referring the tables 1-5 in the text of the manuscript, has been well accepted and we have corrected all the tables in the manuscript text to refer the tables sequentially as they appear in the manuscript text. Thank you!
9. Regarding to check whether retracted articles are still cited in the manuscript, we have checked the references and no retracted reference is used. Thank you!
10. According to the requirement for the ORCID ID, the corresponding author and some of co-authors such as (Amene Abebe) already had ORCID iD. Thank you!
11. The comment about overlapping texts with previous publication is correct. However, nothing was deliberate because the publications which I were a co-author were published in the year 2020 and the current study has been done in the year 2018. Though, we have rephrased the overlapping texts in the current version of the manuscript. Thank you!
B. **Point by point response letter to reviewer one**
1. Concerning the reviewer comment about revision of the document, form and the statistical part. We have re-written the document and corrected all errors in the write up and statistical output reporting
2. The reviewer comment about reduction of the introduction section of the manuscript is well accepted. We have reduced the introduction section to only one page and a paragraph, and restructured the content in to what has been known in the existing literature, what is lacking and what has been aimed by the study. Thank you!
C. **Point by point response letter to reviewer two**

First of all we would like to express our gratefulness for the reviewer for appropriately recognizing the topic as one of the important area of research and for being interested on the topic. Following our acknowledgement, the reviewers concerns are point by point addressed in the following ways:

1. Based on the recommendation of the reviewer to rephrase the title, we have rephrased the title as per the suggestion. Thank you!
2. The comment about writing in full the abbreviations at least once before using the abbreviation is well accepted. We have written all the abbreviation in full at least once before we use the abbreviations in the manuscript file. Thank you!
3. The reviewer comment about erasing unnecessary space between words and punctuation is right. We have erased all the unnecessary spaces and also checked punctuation errors throughout the document and corrected. Thank you!
4. The reviewer comments regarding the structure of the introduction section of the previous version of the manuscript has been accepted. In the current version we re-structured the contents of the introduction in to three sections “introduction” “significance of the study” and “aim of the study” and we have removed all the repeated concepts which unnecessarily lengthened the introduction section of the manuscript. Thank you so much!
5. The reviewer comment about the use of comma in English is not used in maths is right. However, we search throughout the manuscript document and couldn’t get such error. Thank you!
6. The reviewer comment about insertion of references to support ideas is accepted. We have cited the references as needed throughout the manuscript document. Thank you!
7. The reviewer comment regarding the repetition of words like “the zone” and “the region” unnecessarily have been removed and corrected. Thank you!
8. According to the reviewers comment to correct the way of writing of the dates in the methods section is accepted. We have corrected the writing in the current version. Thank you!
9. The reviewer comment regarding rephrasing or inclusion of the map of the study area is right. We have rephrased and restructured the description of the study setting, study design and study period and made more clear and precise. Thank you!
10. The sentence in the methods section which written (The study utilized Facility based …..health workers involved in RHIS activities) has been reformulated. Thank you!
11. The reviewer recommendation to restructure and combine the repeated points in the methods section is well accepted. In the recent version we have removed the repeated points and also combined whose idea is the same. Thank you!
12. The comment of the reviewer about not use the bullet (►) is accepted. We have corrected in the current version. Thank you!
13. The comment about reclassifying of the axes and summarizing the results is accepted. We have summarized and present the results section in a clearer way in this version. Thank you!
14. The reviewers comment about empty boxes in the table 5 is corrected. Thank you!
15. The reviewers comment about small tables is correct and we have combined table 3 and 4.Thank you!
16. The reviewer comment regarding the form of references is not that requested by PLOSONE revue is right and we have corrected in the current version as per the journal reference citation requirement. Thank you!
