# Supplementary material for: Data quality assessment and associated factors in the health management information system among health centers of Southern Ethiopia
Source: PLoS One. 2021 Oct 27;16(10):e0255949. doi: 10.1371/journal.pone.0255949 (PMC8550403; doi:10.1371/journal.pone.0255949)
Supplement: Attachment — Submitted filename: response to reviewers.docx [file pone.0255949.s007.docx]

**Response to academic editors and the reviewer**

First of all, we would like to acknowledge the academic editor for giving us adequate time to revise and address all the concerns of the reviewer and journal requirements. We have addressed the recommendations of the reviewer on the second version of the manuscript as follows. Thank you!

**Response to the academic editors**

- Regarding the journal requirement about the citation of the retracted articles, we have checked the references we used and no retracted article has been cited in in our manuscript. Thank you!

**Response to the reviewer**

1. As per the recommendation of the reviewer to modify the title as “Data quality assessment and associated factors in the Health Management Information System among Health Centers of Southern Ethiopia” is accepted and we have modified it as recommended. Thank you!
2. The reviewer recommendation to add the geographic map of the study area is accepted and we have added the study area map in the “study setting, study design and study period” section of the current revised manuscript. Thank you!
3. According to the reviewer suggestion not to use decimal ”.”and”%” at the same time with in (0.9 or 90% , 1.10 or 110% ) page 13 and page 15. We have corrected the numbers to be reported only with the decimal numbered. Thank you!
4. According to the reviewer recommendation to remove the parenthesis from the percentage page 18, 57.7%. We have removed the parenthesis. Thank you!
5. According to the reviewer suggestion to add”=” in page 23 paragraph 4 [AOR = 3.4, 95% CI (1.3, 8.7)]. We have added the equality sign. Thank you!
6. We would like to appreciate the reviewer for showing repetition of reference 5 and 8 and we have removed reference number 8 and corrected the whole document references sequentially. Thank you!
7. Regarding the reviewer comment to maintain consistency of the same authors naming in reference number 28 and 29, the comment is well accepted and we have made the same style of citation in both references. Thank you!
