# Supplementary material for: Data quality assessment and associated factors in the health management information system among health centers of Southern Ethiopia
Source: PLoS One. 2021 Oct 27;16(10):e0255949. doi: 10.1371/journal.pone.0255949 (PMC8550403; doi:10.1371/journal.pone.0255949)
Supplement: Attachment — Submitted filename: response to reviewers.docx [file pone.0255949.s008.docx]

**Response to academic editors and the reviewer**

First of all, we would like to acknowledge the academic editor for giving us adequate time to make final revision and address all the concerns of the reviewer and journal requirements. We have addressed the recommendations of the reviewer on the third version of the manuscript as follows. Thank you!

**Response to the academic editor**

- Regarding the journal requirement about the citation of the retracted articles, we have checked the references we used and no retracted article has been cited and no changes have been made in the references section from the previous in the current our manuscript. Thank you!

**Response to the reviewer**

1. As per the recommendation of the reviewer to modify reference number 26 in the reference list from “FMoH” to “FMOH”. We have made the recommended revision as per the reviewer recommendation. Thank you!
